# Interactive visualization of clusters in microarray data: an efficient tool for improved metabolic analysis of E. coli

**DOI:** 10.1186/1475-2859-8-37

**Published:** 2009-07-15

**Authors:** Theresa Scharl, Gerald Striedner, Florentina Pötschacher, Friedrich Leisch, Karl Bayer

**Affiliations:** 1 Department of Statistics and Probability Theory, Vienna University of Technology, Wiedner Hauptstr. 8-10, A-1040 Vienna, Austria; 2 Department of Biotechnology, University of Natural Resources and Applied Life Sciences, Vienna, Muthgasse 18, A-1190 Vienna, Austria; 3 Department of Statistics, University of Munich, Ludwigstr. 33, D-80539 Munich, Germany

## Abstract

**Background:**

Interpretation of comprehensive DNA microarray data sets is a challenging task for biologists and process engineers where scientific assistance of statistics and bioinformatics is essential. Interdisciplinary cooperation and concerted development of software-tools for simplified and accelerated data analysis and interpretation is the key to overcome the bottleneck in data-analysis workflows. This approach is exemplified by gcExplorer an interactive visualization toolbox based on cluster analysis. Clustering is an important tool in gene expression data analysis to find groups of co-expressed genes which can finally suggest functional pathways and interactions between genes. The visualization of gene clusters gives practitioners an understanding of the cluster structure of their data and makes it easier to interpret the cluster results.

**Results:**

In this study the interactive visualization toolbox gcExplorer is applied to the interpretation of *E. coli *microarray data. The data sets derive from two fedbatch experiments conducted in order to investigate the impact of different induction strategies on the host metabolism and product yield. The software enables direct graphical comparison of these two experiments. The identification of potentially interesting gene candidates or functional groups is substantially accelerated and eased.

**Conclusion:**

It was shown that gcExplorer is a very helpful tool to gain a general overview of microarray experiments. Interesting gene expression patterns can easily be found, compared among different experiments and combined with information about gene function from publicly available databases.

## Background

The implementation of comprehensive analysis tools from systems biology into bioprocess development concepts enables the change from empirical to rational knowledge based approaches in host engineering and process design. DNA microarrays are powerful, state of the art tools for the monitoring of cellular systems on transcriptome level providing insight into cellular response to defined changes in cultivation conditions, e.g induction of recombinant protein production [1]. The successful application of microarrays as monitoring tool in bioprocess development strongly depends on concerted design of cultivation experiments as well as array experiments and systematic data analysis. To enable interpretation of results the most significant information must be extracted from the acquired microarray data by using optimally suited methods of statistics and bioinformatics. Comparative analysis of data sets from independent experiments provide additional information and contributes to the optimal exploitation of microarray data. Cluster analysis is frequently used in gene expression data analysis to find groups of co-expressed genes which can finally suggest functional pathways and interactions between genes. Clusters of co-expressed genes can help to discover potentially co-regulated genes or genes associated to conditions under investigation, i.e., the induction strategies. Usually cluster analysis provides a good initial investigation of microarray data before actually focusing on smaller gene groups of interest. In the literature numerous cluster algorithms for clustering gene expression data have been proposed. Besides traditional methods like hierarchical clustering, K-means, partitioning around medoids (PAM, K-medoids) or self-organizing maps there are several algorithms dealing with time-course gene expression data (e.g., [2-5]). Clustering is commonly used to reduce the complexity of the data from multidimensional space to a single nominal variable, the cluster membership. In the analysis of microarray data clustering is used as vector quantization because no clear density clusters exist in the data. Genetic interactions are so complex that the definition of gene clusters is not clear. Additionally microarray data are very noisy and co-expressed genes can end up in different clusters. Therefore the set of genes is divided into artificial subsets where relationships between clusters play an important role. Depending on the purpose of the cluster analysis different numbers of clusters can be appropriate. Few large clusters are typically used for a broad overview of a data set and many small clusters are more suitable to detect co-regulated genes (e.g., over 25 clusters in [2]).

The display of cluster solutions particularly for a large number of clusters is very important in exploratory data analysis. Visualization methods are necessary in order to make cluster analysis useful for practitioners. They give an understanding of the relationships between segments of a partition and make it easier to interpret the cluster results. In this work neighborhood graphs [6] are used for visual assessment of the cluster structure of partitioning cluster solutions.

All cluster algorithms and visualization methods used are implemented in the statistical computing environment R ([7], ). R package flexclust[6] contains extensible implementations of the K-centroids and QT-Clust algorithm. The new interactive visualization toolbox gcExplorer[8] uses the non-linear graph layout algorithms implemented in the open-source graph visualization software Graphviz () for the arrangement of nodes. Bioconductor packages graph and Rgraphviz[9] provide tools for creating, manipulating, and visualizing graphs in R as well as an interface to Graphviz. The gcExplorer contains several possibilities to investigate gene clusters. A detailed view of single clusters is given by clicking on the nodes of the graph where various panel functions can be used to show the corresponding genes, e.g., matrix plots for gene expression profiles over time or HTML tables giving detailed information about differential expression as well as links to databases. Properties of the clusters can be included in the display of the neighborhood graph, e.g., cluster size or cluster tightness. Additionally external knowledge from differential expression analysis or functional grouping is used to investigate the data. Finally different experiments can easily be compared by visualizing groups of genes with common expression pattern in one experiment and potentially different expression pattern in the other experiment. The latest release of gcExplorer is always available at the Comprehensive R Archive Network CRAN: .

In this paper the utility of the interactive visualization toolbox gcExplorer is demonstrated for the interpretation of *E. coli *microarray data. The data sets used derive from two independent fedbatch experiments conducted in order to investigate the impact of different induction strategies on the host metabolism and product yield. The goal of the comparison is to identify genes and pathways that act similar in both settings and more importantly to identify groups of genes with differential reaction to the two induction strategies. For this reason cluster analysis followed by comparative graphical investigation of the different groups of genes is performed. The graphical exploration of clusterings is applicable to arbitrary partitioning cluster solutions. In this case the stochastic quality cluster algorithm QT-Clust [10] is used. In the Methods Section this cluster algorithm and the concept of neighborhood graphs are reviewed for completeness. The data sets used are described in the Data Section. In the Results Section several steps of the analysis of the given data sets are presented including the visualization of the cluster structure and the direct graphical comparison of these two experiments. Further, a method is presented how to include external knowledge about gene function in the display of cluster solutions. It is shown that the identification of potentially interesting gene candidates or functional groups is substantially accelerated and eased.

## Methods

### Cluster Algorithm

In this paper the quality-based cluster algorithm stochastic QT-Clust [10] is used which is an adaptation of the original QT-Clust algorithm proposed by Heyer et al. [2]. In contrast to cluster algorithms like K-means where the number of clusters is defined a priori the quality of clusters is the central parameter now. The quality of a cluster is given by the maximum diameter of the cluster. The possibility to tune the quality of clusters is very helpful for practitioners. Depending on the goal of the experiment different properties of the clusters are desirable which can either be a few rather large clusters or many small clusters with very specific expression patterns. Additionally the minimum number of points that form a single cluster is chosen. Microarray data are noisy data and outliers can easily distort cluster solutions. Stochastic QT-Clust is robust to outliers as outlier observations will not be added to any cluster. Hence the number of clusters is controlled indirectly through these two parameters. A further tuning parameter is the number ntry of candidate clusters generated in each run. The algorithm works as follows:

1. Start with a randomly chosen centroid.

2. Iteratively add the gene that minimizes the increase in cluster diameter.

3. Continue until no gene can be added without surpassing the diameter threshold.

4. Repeat from 1. for ntry - 1 further centroids.

5. Select the largest candidate cluster and remove the genes it contains from further consideration.

6. Goto 1. on the smaller data set.

7. Stop when the largest remaining cluster has fewer than some prespecified number of elements.

If ntry is equal to the number of genes *G *the original QT-Clust algorithm is obtained. Stochastic QT-Clust speeds up the procedure and yields different local maxima of the objective function. The original algorithm will always converge in the same local optimum.

In order to gain maximum information the choice of the cluster diameter and the minimum number of points has to be carefully chosen as both have a large impact on the resulting clustering and its interpretation. A small diameter will yield a cluster solution with many small clusters containing genes with very similar expression patterns whereas a larger diameter will result in a smaller number of less tight clusters. Additionally, if the diameter is chosen too small many genes cannot be added to a cluster and will be treated as outliers. The minimum number of points also has a big in influence on the number of clusters and the number of outliers. If small clusters are allowed (e.g., the minimum number of points is 2) there will be less outliers than in the case of a larger minimum number of points. There is a tradeoff between the number of clusters, the size of the clusters and the number of outliers. Therefore it is necessary to finetune these parameters for each data set to obtain a cluster solution that fits the needs of the current experiment. In order to use the neighborhood graph for the visualization of a cluster solution obtained from QT-Clust the corresponding cluster centroids are computed. However, neighborhood graphs are generally applicable to various partitioning cluster algorithms like the well-known K-means or PAM.

### Neighborhood Graphs

Neighborhood graphs [6] use the mean relative distances between points as edge weights in order to measure how separated pairs of clusters are. Hence they display the distance between clusters. In the graph each node corresponds to a cluster centroid and two nodes are connected by an edge if there exists at least one point that has these two as closest and second-closest centroid.

For a given data set *X*_*N *_= {*x*_1_,...,*x*_*N*_} the distance between points *x*_*i *_and *x*_*j *_is given by *d*(*x*_*i*_, *x*_*j*_), e.g., the Euclidean or absolute distance. *C*_*K *_= {*c*_1_,...,*c*_*N*_} is a set of centroids and the centroid closest to *x *is denoted by



The second closest centroid to *x *is denoted by



The set of all points where *c*_*k *_is the closest centroid is given by



Now the set of all points where *c*_*i *_is the closest centroid and *c*_*j *_is second-closest is given by



For each observation *x s*(*x*) is defined as



*s*(*x*) is small if *x *is close to its cluster centroid and close to 1 if it is almost equidistant between the two cluster centroids. The average s-value of all points where cluster *i *is closest and cluster *j *is second closest can be used as a proximity measure between clusters and as edge weight in the graph.



|*A*_*i*_| is used in the denominator instead of |*A*_*ij*_| to make sure that a small set *A*_*ij *_consisting only of badly clustered points with large *s*-values does not induce large cluster similarity.

Neighborhood graphs are a useful tool for the visualization of the structure of a cluster solution. Additionally they can be used as exploratory tool to determine the quality of a given clustering and to validate the number of clusters.

### Data

**Figure 1:**
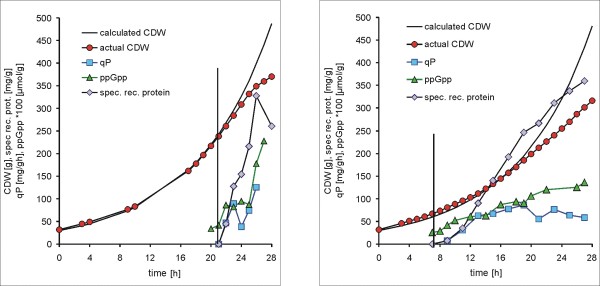
**Process data**. Protein production process with *E. coli *HMS174(DE3)(pET30aNProGFP). Fully induced system (experiment A, left panel) and partially induced system (experiment B, right panel). Vertical line indicates time point of induction.

The *E. coli *cultivation data were collected at the Department of Biotechnology at the University of Natural Resources and Applied Life Sciences in Vienna. Two recombinant *E. coli *processes with different induction strategies were conducted in order to evaluate the in influence of the expression level of the inclusion body forming protein N^*pro*^GFPmut3.1 on the host metabolism. The standard strategy with a single pulse of inducer yielding in a fully induced system (in the following called experiment A) was compared to a process with continuous supply of limiting amounts of inducer resulting in a partially induced system (in the following called experiment B) [11]. The time point of induction of the partially induced system was set one doubling past feed start. The bioreactor, the used equipment as well as the on-and offline analysis was published in detail by Achmüller et al. [12]. The resulting process data shown in Figure 1 clearly emphasize the central impact of induction strategies on the cellular response of strong expression systems and their behavior in production processes. The product formation rate triggered by full induction is too high and the thereby provoked metabolic overload impedes cellular growth. The increase in the total cell dry weight (CDW) attained past induction was mainly caused by the formation of the recombinant protein. This means that growth and product formation were decoupled completely. In consequence of these reactions product formation and process control were maintained only for a short period. However, in the experiment with limited induction cells were able to cope with the metabolic load triggered by the recombinant gene expression level for more than one doubling. Product formation was tightly coupled to cellular growth but approximately 9 hours past induction the metabolic load level exceeded the cellular capacities. The glucose yield coefficient (*Y*_*X*/*S*_) decreased and the cells lost their ability to divide. The net cell mass generated in this phase was channeled into cell size and the cells entered a similar state as in the process with full induction.

In order to analyze the cellular response to different induction strategies on the transcription level two independent DNA microarray experiments were performed. A dye-swap design was used and the cells in the non-induced state of each experiment were compared to samples past induction. Since the production period of the fully induced system was limited to approximately one generation (7 h at a growth rate of 0.1 h^-1^) samples were drawn in a frequency of 1 h^-1^. To cover the production period of the process with limited induction the sampling frequency was reduced to one sample every two hours. The used microarrays were epoxide-coated slides (Corning^® ^Epoxide Coated Slides) with selective probes (50-mer oligos) for all 4289 open reading frames of the *E. coli *K12 genome (MWG *E. coli *K12 V2 oligo set; MWG Biotech AG, Germany) spotted in duplicates. The two experiments (including all processing protocols) have been loaded into ArrayExpress (). The ArrayExpress accession number of the array design is A-MARS-10. The experiment with fully induced *E. coli *expression system (experiment A) has accession number E-MARS-16 and the experiment with partially induced system (experiment B) has accession number E-MARS-17. For standard low level analysis the data were preprocessed using print-tip loess normalization. Differential expression estimates were calculated using Bioconductor [13]() package limma[14]. The two data sets were filtered by excluding genes expressed at a very low level (average log_2 _intensity smaller 8), genes not showing differential expression (log-ratio M smaller ± 1.5) at least at one time point and genes with p-value of the corresponding F-statistic smaller 0.05. After filtering the data acquired from the experiment with a fully induced *E. coli *expression system (experiment A) consists of 733 genes and the data acquired from the process with limited induction (experiment B) consists of 429 genes where 311 genes are differentially expressed in both experiments. The filtered data sets were clustered using stochastic QT-Clust and further analysis and visualization was conducted using the gcExplorer.

## Results

### Cluster Visualization and Interpretation

The major goal of this study is to identify differences between two independent microarray experiments which cannot be compared directly. For this purpose the two data sets are clustered into small and tight subgroups of genes with common expression pattern which can easily be investigated. The diameter of the clusters is tuned in such a way to get in the range of 15 clusters and 10 outliers. The minimum number of points that form a single cluster is set to 2. These parameter settings lead reasonable cluster solutions that can directly be interpreted. The data sets of experiments A and B were separated into 19 and 15 clusters respectively with 20 and 9 outliers. Next these two cluster solutions are investigated independently and combined in the following section. In case of very similar clusters the neighborhood graph can be used to combine the clusters after proofing the similarity. However, in this exploratory approach it is advantageous to merge similar clusters than to split large ones.

**Figure 2:**
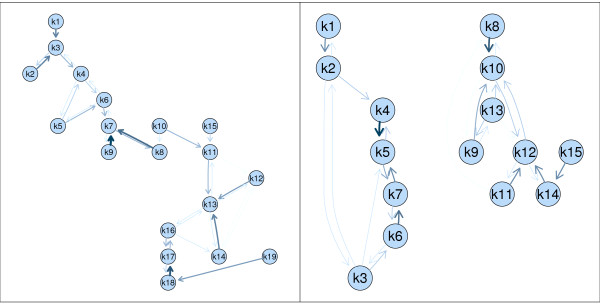
**Neighborhood graphs**. Neighborhood graph of the QT-Clust cluster solutions for experiment A (left panel) and experiment B (right panel).

**Figure 3:**
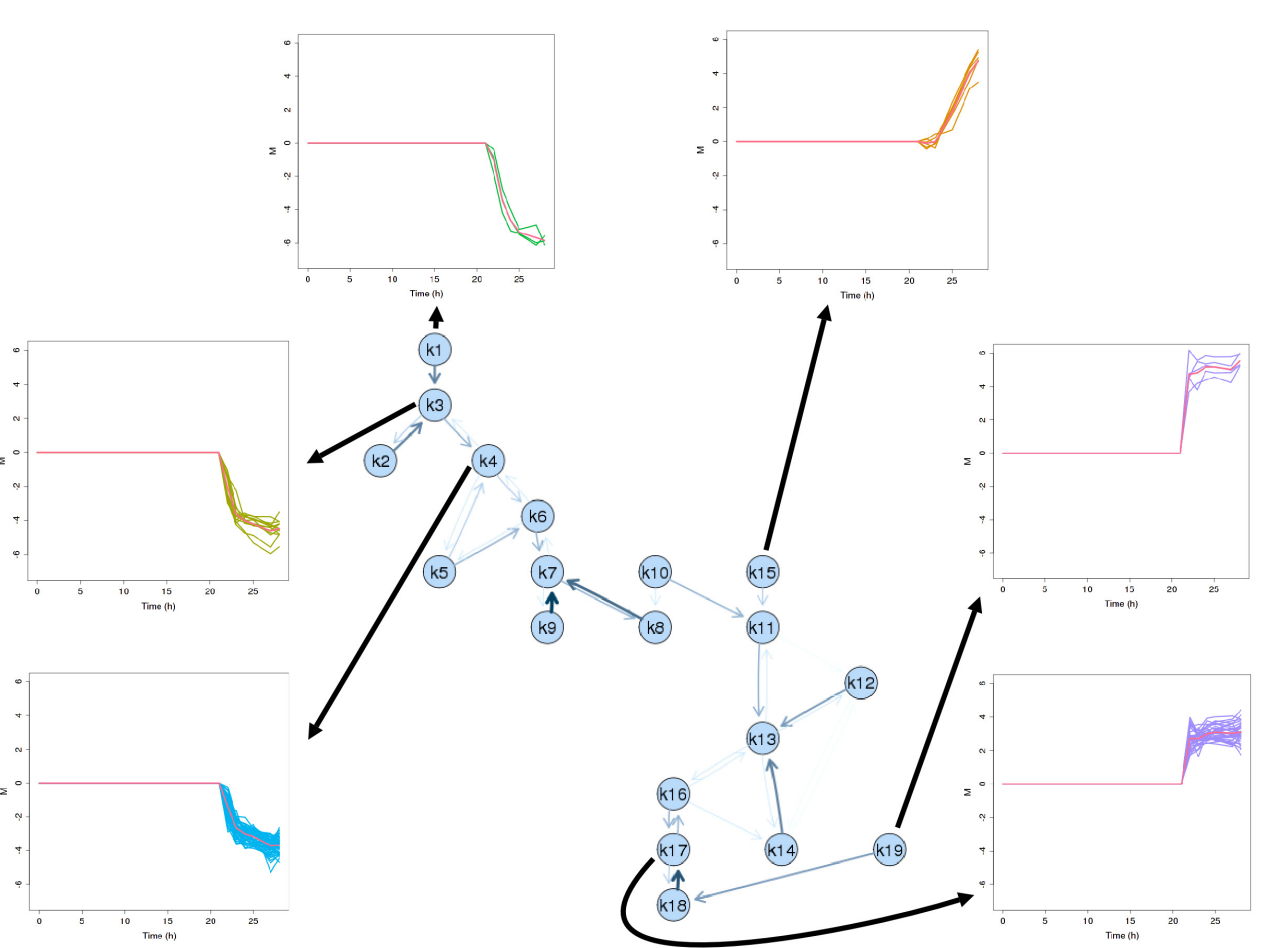
**Diagram of experiment A**. Neighborhood graph of experiment A with selected gene expression profiles displayed.

The resulting cluster solutions are visualized as neighborhood graphs in Figure 2 using the gcExplorer where nodes correspond to cluster centroids. In the two graphs relationships between clusters can easily be explored as similar clusters are connected by edges. The thicker and darker an edge is drawn the more similar two clusters are. Several groups of clusters can be found. In the neighborhood graph of experiment A the clusters in the top left corner (e.g., 1,2,3) are not connected to the clusters in the bottom right corner (e.g., 17,18,19) indicating that the corresponding genes show very different expression profiles. This can be confirmed by looking at the expression profiles of the corresponding genes of experiment A (see Figure 3). The genes in the bottom right clusters are all up-regulated (e.g., clusters 17 and 19) whereas the genes in the top left clusters are down-regulated (e.g., clusters 1, 3, and 4). The obtained results clearly show that the information gain of this work benefits from splitting the data sets in many small clusters at the beginning. For example, cluster 17, 18 and 19 contain genes with similar expression profiles. However, the level of up-regulation is much higher in cluster 19. If interpretation of a general trend is required these small clusters can be treated as a large one as it is often easier to investigate the smaller ones.

The cluster profiles with immediate and stern up or down regulation followed by constant values for the rest of the process definitely reflect the macroscopic outcome of the experiment with full induction. The irreversibility of the cellular response to the applied load level is mirrored in the transcriptome data. The only exception are the transcription profiles of genes related to phage shock grouped in cluster 15 which show continuously increasing gene expression until the end of the process.

**Figure 4:**
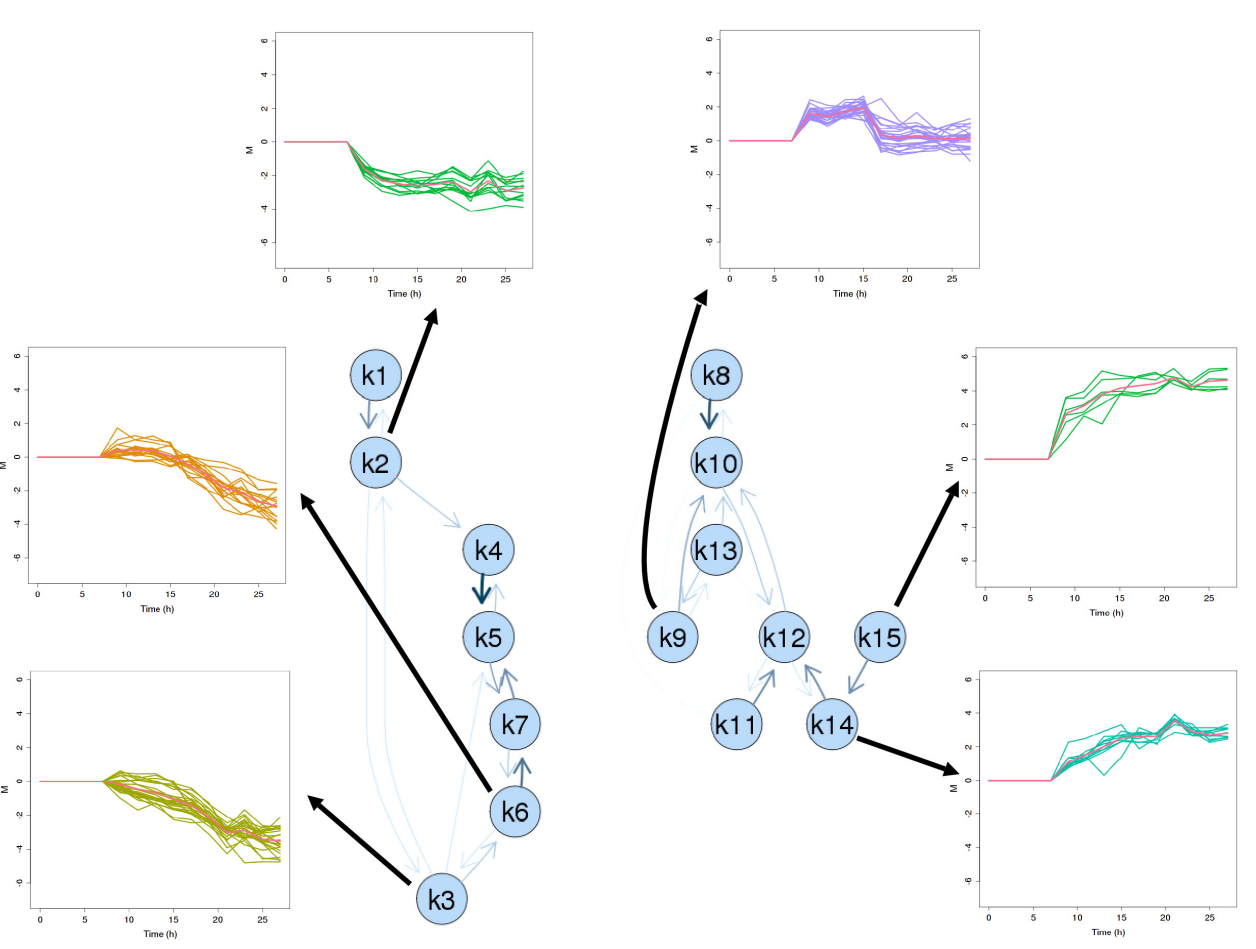
**Diagram of experiment B**. Neighborhood graph under limited conditions (experiment B) with selected gene expression profiles displayed.

A more detailed view on the cluster solution of experiment B is given in Figure 4. The neighborhood graph of this cluster solution consists of two unconnected subgraphs and shows a higher degree of differentiation. The subgraph on the left contains all down-regulated genes whereas the subgraph on the right contains all clusters with up-regulated genes. Beside clusters with direct response to induction (e.g cluster 2 and 15) additional cluster profiles with immediate up-regulation followed by a downregulation after 9 hours past induction (e.g. cluster 9 and 13) or profiles with a 9 hour-delayed response to induction (e. g. cluster 6 and 3) were obtained. Furthermore, a large number of genes belongs to clusters with continuously increasing or decreasing trends past induction. These findings distinctly contradict the results of experiment A where only few genes show such a behavior. Again, the transcription data precisely reproduce the major changes in the experiment, the induction and the incipient metabolic overload. The new visualization toolbox offers various possibilities for the analysis of microarray data which cannot all be shown here. In the graphs shown so far simple node symbols are used including the number of the corresponding cluster but there are several possibilities how to include additional information in the representation of nodes. The most simple method is to use color coding, e.g., to color nodes by size or tightness of the corresponding clusters. Another possibility is to use different shapes or symbols for nodes representing clusters with specific properties. The neighborhood graph is implemented in an interactive way and gene clusters can be investigated by clicking on the nodes. Plots of the expression profiles of the corresponding genes pop up and HTML tables giving further information about the genes link to databases like Ecocyc (). The gcExplorer is applicable up to a very high number of clusters. Related clusters are not forced to lie next to each other in the graph as edges can have various lengths (e.g., the edge between clusters 18 and 19 in the left panel or the edge between clusters 2 and 3 in the right panel).

### Functional Grouping

Cluster analysis is used to find groups of co-regulated genes in the microarray data without prior knowledge about the gene functions. However, by clustering expression profiles of co-expressed genes groups of genes with similar function are found. External information about the annotation of genes to functional groups can easily be included in the neighborhood graph, e.g., the accumulation of gene ontology (GO, [15]) classifications in certain gene clusters can be highlighted in the node representation. For *E. coli *GO classifications about biological process (GOBP), molecular function (GOMF) and cellular component (GOCC), the GenProtEC ([16], ) classification system for cellular and physiological roles of *E. coli *gene products and the RegulonDB ([17], ) providing information about operons and regulatory networks were implemented. These knowledge-based functional mappings can be used to study cellular functions in individual clusters.

**Figure 5:**
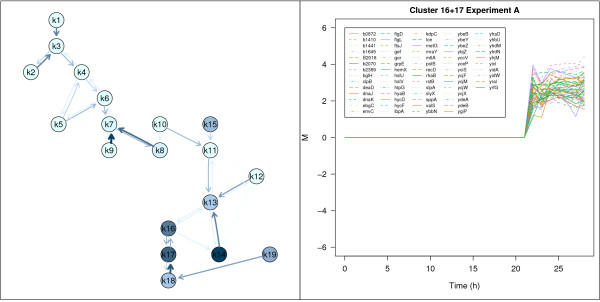
***σ*^32^-regulated genes**. Neighborhood graph of experiment A where clusters containing *σ*^32^-regulated genes are highlighted (left panel). Gene expression profiles of the corresponding genes of clusters 16 and 17 are shown in the right panel.

In the left panel of Figure 5 clusters of experiment A with genes controlled by *σ*^32^, the main regulator of heat shock response are highlighted. In the right panel gene expression profiles of the closely related clusters 16 and 17 are displayed. 21 of 66 genes of the two clusters are under control of *σ*^32^. Further functional characterization of these two clusters using GOMF yields the assignment of 26 genes to the GO-term GO:0005515 (protein binding) and of 16 genes to GO:0005524 (ATP binding). GOBP maps 11 genes to GO:0006950 (response to stress) and 10 genes to GO:0006457 (protein folding). On the other hand, a considerable number of 18 genes of these clusters is not mapped by the GO classification system as their molecular function is unknown or uncertain. Their cluster membership provides hints how these genes are embedded in the regulatory network of the cell and suggests potential cellular functions. A good example is *ybb*N, a thioredoxin-like protein with chaperone properties recently demonstrated in in-vitro experiments [18-20]. The relevance of the thus determined properties for cell physiology is still unknown but the cluster result strongly supports the suggested function as chaperone. Construction of a *ybb*N deletion mutant, a clone with plasmid encoding *ybb*N and conduction of experiments similar to the described cultivations will provide the information which is required to confirm these assumptions.

### gcExplorer – a tool for comparative graphical analysis of microarray experiments

**Figure 6:**
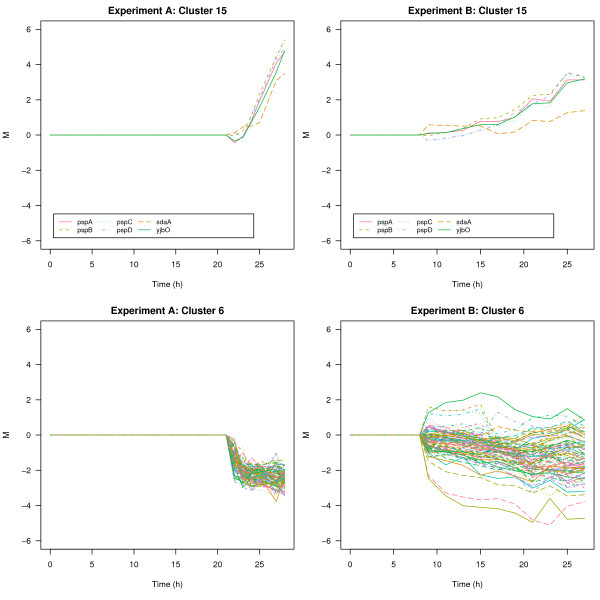
**Comparative graphical analysis of microarray experiments**. Comparison of cluster 15 and 6 of experiment A under fully induced conditions (left panels) and limited conditions (right panels).

One typical application of the gcExplorer is the comparative graphical analysis of different and independent *μ*-array experiments. It is exemplified in the following workflow. A cluster solution of a single experiment (e.g., experiment A) can easily be compared to other experiments (e.g., experiment B) in order to find genes or groups of genes with similar as well as different behavior. This is achieved by clustering the genes of experiment A and using this partition to investigate experiment B. This procedure helps to quickly identify groups of genes that cluster in both experiments and on the other hand to reveal differences between the experiments. An example of a gene cluster which is very similar between the two experiments is shown in the top panels of Figure 6. In the top left panel cluster 15 of experiment A is shown for the full induction data. In the top right cluster the same set of genes is shown under limited conditions. An example of a tight cluster of genes showing a strong and direct down-regulation in response to induction is given by cluster 6 (see Figure 6 bottom left panel). In experiment B the majority of genes grouped to cluster 6 of experiment A show a delayed rather than a direct down regulation in response to induction (bottom right panel of Figure 6) whereas a considerable number of genes shows no common behavior. In the following *fli*E, *fli*A and *lpp*, three genes with deviating profiles were selected to be examined in more detail.

**Figure 7:**
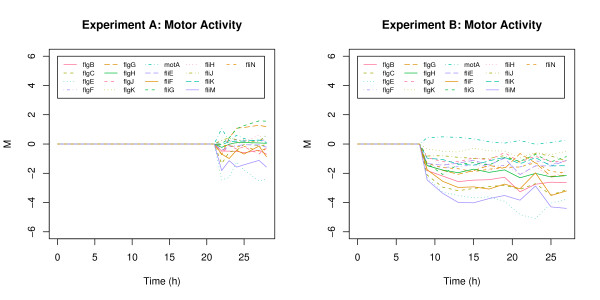
**Motor Activity**. Motor Activity (GO:0003774) in experiment A (left panel) and experiment B (right panel).

*fli*A and *flg*E are the only genes of cluster 6 showing strong and direct down-regulation under limited induction conditions. These genes belong to the GO group GO:0003774 (motor activity). The expression patterns of all genes of this group are shown in Figure 7. In the experiment with limited induction (right panel) all these genes were down regulated in contrast to experiment A (left panel) where no common response was detected. A possible explanation of these findings is that cells exposed to high but tolerable induction levels (experiment B) were able to compensate for depletion of cellular resources and capacities by reduction or cessation of non essential branches of the metabolism. In the defined environment of a bioreactor motility provides no benefits but demands energy and metabolites. Consequently, the cells cut down these expenses to maintain central cellular functionality.

**Figure 8:**
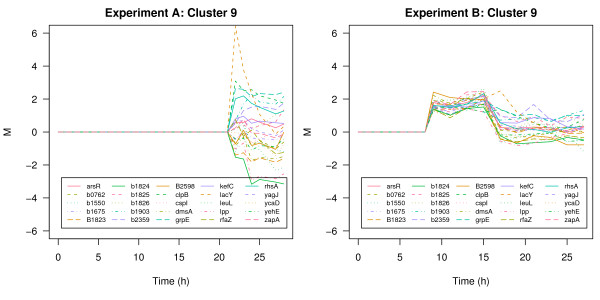
**Different response: the lpp cluster**. Cluster 9 of experiment B under fully induced conditions (left panel) and limited conditions (right panel).

Another interesting transcription profile in cluster 6 of experiment A is given by the murein lipoprotein *lpp*. Under fully induced conditions this gene is down-regulated whereas under limited induction conditions the expression level of this gene follows a distinct up- and down trend coinciding with the process phenomenons described in Section Data. In the cluster solution of experiment B *lpp *is assigned to a cluster of 25 genes (Figure 8) comprising genes involved in membrane lipid synthesis (*gns*B, *rfa*Z), membrane sysnthesis (*rhs*A), cell division (*zap*A) cold shock response (*csp*BCI) but also 8 predicted genes of unknown function. Lpp is the major lipoprotein of the outer membrane and one of the most abundant proteins in *E. coli*. It is essential for the stabilization and integrity of the bacterial cell envelope [21]. The *gns*B gene increases the membrane fluidity and flexibility [22]. Cells activate an energy demanding protective strategy by synthesis and translocation of Lpp which is in contrast to the cut-back strategy described above. This comparative analysis of the two experiments clearly reveals the irreversible overload of metabolism in the experiment with full induction. Cells were not able to respond in a concerted and accurate way. On the other hand, cells exposed to limited induction of recombinant gene expression cope with emerging stress by different strategies in order to survive. The described cellular responses are similar to transitional changes of cells entering the stationary phase. This spore-like multiple-stress resistance state enables maintenance of viability under bad conditions [23]. The identified genes involved in these defense mechanisms are potential candidates for indepth investigation and provide clues about the regulatory mechanisms involved.

## Conclusion

The interactive visualization tool gcExplorer was developed in order to make cluster analysis useful for practitioners. It allows not only to visualize the cluster structure, beyond that the gene clusters are plotted or shown in HTML tables with links to databases. Additional properties of the clusters like cluster size or cluster tightness can be highlighted as well as external information like functional grouping. Furthermore gcExplorer provides functions for comparative graphical analysis of different *μ*-array experiments. gcExplorer is a userfriendly software tool for the analysis of gene expression data and very helpful for practitioners to get an overview on the output of *μ*-array experiments.

In this study microarray data from two processes with a strong recombinant *E. coli *expression system were analyzed. Neighborhood graphs enable the investigation of the underlying cluster structure and relationships between clusters. The implemented features for functional grouping allowed the assignment of cellular functions to clusters and provided hints about the functionality of other genes belonging to a certain cluster. Comparative graphical analysis of these two experiments resulted in the identification of differences in the cellular response and a number of interesting gene candidates involved. It was shown that the cellular strategies are different in the two DNA-*μ*-array experiments. Useful information was extracted for the further advancement of the expression system by means of genetic engineering or by means of process engineering.

## Competing interests

The authors declare that they have no competing interests.

## Authors' contributions

TS and GS contributed equally to this manuscript. TS and GS carried out the analysis of the data and wrote the manuscript. TS implemented the software. GS and FP performed the experimental part of the work. All authors participated in design and discussion of the research. FL and KB directed the research.
